# Mathematical Approach to Differentiate Spontaneous and Induced Evolution to Drug Resistance During Cancer Treatment

**DOI:** 10.1200/CCI.18.00087

**Published:** 2019-04-10

**Authors:** James M. Greene, Jana L. Gevertz, Eduardo D. Sontag

**Affiliations:** ^1^ Rutgers University, New Brunswick, NJ; ^2^ The College of New Jersey, Ewing Township, NJ; ^3^ Northeastern University, Boston, MA; ^4^ Harvard Medical School, Cambridge, MA

## Abstract

**Purpose:**

Drug resistance is a major impediment to the success of cancer treatment. Resistance is typically thought to arise from random genetic mutations, after which mutated cells expand via Darwinian selection. However, recent experimental evidence suggests that progression to drug resistance need not occur randomly, but instead may be induced by the treatment itself via either genetic changes or epigenetic alterations. This relatively novel notion of resistance complicates the already challenging task of designing effective treatment protocols.

**Materials and Methods:**

To better understand resistance, we have developed a mathematical modeling framework that incorporates both spontaneous and drug-induced resistance.

**Results:**

Our model demonstrates that the ability of a drug to induce resistance can result in qualitatively different responses to the same drug dose and delivery schedule. We have also proven that the induction parameter in our model is theoretically identifiable and propose an in vitro protocol that could be used to determine a treatment’s propensity to induce resistance.

## INTRODUCTION

Tumor resistance to chemotherapy and targeted drugs is a major cause of treatment failure. Both molecular and microenvironmental factors have been implicated in the development of drug resistance.^1^ As an example of molecular resistance, upregulation of drug efflux transporters can prevent sufficiently high intracellular drug accumulation, which limits treatment efficacy.^2^ Other molecular causes of drug resistance include modification of drug targets, enhanced DNA damage repair mechanisms, dysregulation of apoptotic pathways, and the presence of cancer stem cells.^1-5^ Irregular tumor vasculature that results in inconsistent drug distribution and hypoxia is an example of a microenvironmental factor that impacts drug resistance.^6^ Other characteristics of the tumor microenvironment influencing drug resistance include regions of acidity, immune cell infiltration and activation, and the tumor stroma.^1,6-10^ Experimental and clinical research continues to shed light on the multitude of factors that contribute to cancer drug resistance. Mathematical modeling studies have also been used to explore both broad and detailed aspects of cancer drug resistance, as reviewed previously.^11-13^

**CONTEXT**

**Key Objective**
  Resistance to chemotherapy may arise from Darwinian selection of resistant subclones that either predate therapy or emerge during treatment. In addition, treatment itself may induce genetic or epigenetic variation that catalyzes drug resistance. This work aims to mathematically tease out these various factors.
**Knowledge Generated**
  A mathematical model is introduced to distinguish the effect of drugs that merely select from those that both create variation and select. The ability of a drug to induce resistance can result in qualitatively different responses on the basis of dose and delivery; constant-infusion regimes are less successful in controlling tumor growth than pulsed therapy for drugs that induce resistance, but the situation is reversed for drugs that act only by selection.
**Relevance**
  Recent experimental evidence suggests that progression to drug resistance need not occur randomly, but instead may be induced by the treatment itself. Understanding the clinical implications of treatment-induced resistance will help formulate appropriate protocols.

Resistance to cancer drugs can be classified as either pre-existing or acquired.^1^ Pre-existing—or intrinsic—drug resistance describes the case in which a tumor contains a subpopulation of drug-resistant cells at the initiation of treatment, which makes therapy eventually ineffective as a result of resistant cell selection.^1^ As examples, pre-existing BCR-ABL kinase domain mutations confer resistance to the tyrosine kinase inhibitor imatinib in patients with chronic myeloid leukemia,^14,15^ and pre-existing MEK1 mutations confer resistance to BRAF inhibitors in patients with melanoma.^16^ Many mathematical models have considered how the presence of such pre-existing resistant cells impacts cancer progression and treatment.^17-40^

Acquired drug resistance broadly describes the case in which drug resistance develops during the course of therapy from a population of cells that were initially drug sensitive.^1^ The term acquired resistance is really an umbrella term for two distinct phenomena, which complicates the study of acquired resistance. On the one hand, there is resistance that is spontaneously—or randomly—acquired during the course of treatment, be it as a result of random genetic mutations or stochastic nongenetic phenotype switching.^41^ This spontaneous form of acquired resistance has been considered in many mathematical models.^18-22,27-29,31,32,35,40,42-49^ On the other hand, drug resistance can be induced (ie, caused) by the drug itself.^41,50-52^

The question of whether resistance is an induced phenomenon or predates treatment was first famously studied by Luria and Delbrück^53^ in the context of bacterial (*Escherichia coli*) resistance to a virus (T1 phage). In particular, Luria and Delbrück hypothesized that if selective pressures imposed by the presence of the virus induce bacterial evolution, then the number of resistant colonies formed in their plated experiments should be Poisson distributed and thus have an approximately equal mean and variance. What Luria and Delbrück found instead was that the number of resistant bacteria on each plate varied drastically, with variance being significantly larger than the mean. As a result, the authors concluded that bacterial mutations predated the viral challenge.^53^

In the case of cancer, there is strong evidence that at least some drugs have the ability to induce resistance, as genomic mutations can be caused by cytotoxic cancer chemotherapeutics.^54,55^ For instance, nitrogen mustards can induce base substitutions and chromosomal rearrangements, topoisomerase II inhibitors can induce chromosomal translocations, and antimetabolites can induce double-stranded breaks and chromosomal aberrations.^54^ Such drug-induced genomic alterations would generally be nonreversible. Drug resistance can also be induced at the epigenetic level.^41,50,56^ For example, expression of multidrug resistance 1 (MDR1), an ABC-family membrane pump that mediates the active efflux of the drug, can be induced during treatment.^1,41^ In another recent example, the addition of a chemotherapeutic agent is shown to induce, through a multistage process, epigenetic reprogramming in patient-derived melanoma cells.^56^ Resistance developed in this way can occur quite rapidly and can often be reversed.^41,52,57^

Despite these known examples of drug-induced resistance, differentiating between drug-selected and drug-induced resistance is nontrivial. For example, what appears to be drug-induced acquired resistance may simply be the rapid selection of a small number of pre-existing resistant cells or the selection of cells that spontaneously acquired resistance.^41,44^ In pioneering work by Pisco and colleagues,^41^ the relative contribution of resistant cell selection versus drug-induced resistance was assessed in an experimental system that involved HL60 leukemic cells that were treated with the chemotherapeutic agent vincristine. After 1 to 2 days of treatment, expression of MDR1 was demonstrated to be predominantly mediated by cell-individual induction of MDR1 expression and not by the selection of MDR1-expressing cells.^41,58^ In particular, these cancer cells exploit their heritable, nongenetic phenotypic plasticity—by which one genotype can map onto multiple stable phenotypes—to change their gene expression to a temporarily more resistant state in response to treatment-related stress.^41,58^

Although there is a wealth of mathematical research that addresses cancer drug resistance, relatively few models have considered drug-induced resistance. Of the models of drug-induced resistance that have been developed, many do not explicitly account for the presence of the drug. Instead, it is assumed that these models apply only under treatment,^41,59-62^ with the effects of the drug implicitly captured in model terms. As these models of resistance induction are dose independent, they are unable to capture the effects that the alteration of the drug dose has on resistance formation. To our knowledge, there have been less than a handful of mathematical models developed in which resistance is induced by a drug in a dose-dependent fashion.^33,34,63^^,^ In Gevertz et al^33^ and follow-up work in Shah, Rejniak, and Gevertz^38^ and Perez-Velazquez et al,^64^ duration and intensity of drug exposure determines the resistance level of each cancer cell. This model allows for a continuum of resistant phenotypes, but is computationally complex as it is a hybrid discrete-continuous, stochastic spatial model. While interesting features about the relationship between induced resistance and the microenvironment have been deduced from this model, its complexity does not allow for general conclusions to be drawn about dose-dependent resistance induction.

Another class of models that addresses drug-induced resistance is that in Chisholm et al.^63^ These models are distinct in that they are motivated by in vitro experiments in which a cancer drug transiently induces a reversible resistant phenotypic state.^52^ The individual-based and integro-differential equation models developed consider rapidly proliferating drug-sensitive cells, slowly proliferating drug-resistant cells, and rapidly proliferating drug-resistant cells. An advection term—with the speed depending on drug levels—is used to model drug-induced adaptation of the cell proliferation level, and a diffusion term for both the level of cell proliferation and survival potential (response to drug) is used to model nongenetic phenotype instability.^63^ Through these models, the contribution of nongenetic phenotype instability (both drug induced and random), stress-induced adaptation, and selection can be quantified.^63^

Finally, the work in Liu et al^34^ models the evolutionary dynamics of the tumor population as a multitype nonhomogeneous continuous time birth-death stochastic process. This model accounts for the ability of a targeted drug to alter the rate of resistant cell emergence in a dose-dependent manner. The authors specifically considered cases in which the rate of mutation that gives rise to a resistant cell: (1) increases as a function of drug concentration, (2) is independent of drug concentration, and (3) decreases with drug concentration. Interestingly, this model led to the conclusion that the optimal treatment strategy is independent of the relationship between drug concentration and the rate of resistance formation. In particular, the authors found that resistance is optimally delayed using a low-dose continuous treatment strategy coupled with high-dose pulses.

As in vitro experiments have demonstrated that treatment response can be affected by drug-induced resistance,^41,52^ in the current work we seek to understand this phenomenon further using mathematical modeling. The initial mathematical model that we have developed—and that will be analyzed herein—is a system of two ordinary differential equations with a single control representing the drug. We intentionally chose a minimal model that would be amenable to analysis, as compared with previously developed models of drug-induced resistance which are significantly more complex.^33,38,63,64^ Despite the simplicity of the model, it incorporates both spontaneous and drug-induced resistance.

In addition to drug-induced resistance, the other characteristic of cancer dynamics we explore is that of traditional, maximally tolerated dose (MTD) treatment protocols compared with high-frequency, low-dose so-called metronomic therapies. Indeed, the differential response between these therapies is fundamentally related to intratumoral heterogeneity/competition, and is explicitly considered in our model. Furthermore, results presented in this work support recent evidence that promotes the adoption of metronomic therapy in many circumstances,^65-69^ and a main objective of this work is to relate competition and drug-induced resistance to therapy design.

This work is organized as follows. We begin by introducing a mathematical model to describe the evolution of drug resistance during treatment with a theoretical resistance-inducing—and noninducing—drug. We use this mathematical model to explore the role played by the drug’s resistance induction rate in treatment dynamics. We demonstrate that the induction rate of a theoretical cancer drug could have a nontrivial impact on the qualitative responses to a given treatment strategy, including tumor composition and the time horizon of tumor control. In our model, for a resistance-preserving drug—that is, a drug that does not induce resistance—better tumor control is achieved using a constant therapeutic protocol versus a pulsed one. Conversely, in the case of a resistance-inducing drug, pulsed therapy prolongs tumor control longer than constant therapy as a result of sensitive/resistant cell competitive inhibition. Once the importance of induced resistance has been established, we demonstrate that all parameters in our mathematical model are identifiable, meaning that it is theoretically possible to determine the rate at which drug resistance is induced for a given treatment protocol. As this theoretical result does not directly lend itself to an experimental approach for quantifying the ability of a drug to induce resistance, we also describe a potential in vitro experiment for approximating this ability utilizing constant therapies. We end with some concluding remarks and a discussion of potential extensions of our analysis, such as a model that differentiates between reversible and nonreversible forms of resistance.

## MATERIALS AND METHODS

Here we introduce a general modeling framework to describe the evolution of drug resistance during treatment. Our model captures the fact that resistance can result from random events or can be induced by the treatment itself. Random events that can confer drug resistance include genetic alterations—for example, point mutations or gene amplification—and phenotype switching.^41^ These spontaneous events can occur either before or during treatment. Drug-induced resistance is resistance specifically activated by the drug and, as such, depends on the effective dose encountered by a cell. Such a formulation allows us to distinguish the contributions of both drug-dependent and drug-independent mechanisms, as well as any dependence on pre-existing—that is prior to treatment—resistant populations.

We consider the tumor to be composed of two types of cells, sensitive (*S*) and resistant (*R*). Sensitive (or wild-type) cells are fully susceptible to treatment, whereas treatment affects resistant cells to a lesser degree. To analyze the role of both random and drug-induced resistance, we use a system of two ordinary differential equations to describe the dynamics between the *S* and *R* subpopulations:

$$\frac{dS}{dt}=r\left(1-\frac{S+R}{K}\right)S-\left(\epsilon +\alpha u\left(t\right)\right)S-du\left(t\right)S+\gamma R,$$ (1)

$$\frac{dR}{dt}=r_{R}\left(1-\frac{S+R}{K}\right)R+\left(\epsilon +\alpha u\left(t\right)\right)S-d_{R}u\left(t\right)R-\gamma R.$$ (2)

All parameters are non-negative. In the absence of treatment, we assume that the tumor grows logistically, with each population contributing equally to competitive inhibition. Phenotypes *S* and *R* each possess individual intrinsic growth rates, and we make the assumption in the remainder of the work that $0\leq r_{R}<r$. This simply states that resistant cells grow slower than nonresistant cells, an assumption that is supported by experimental evidence.^70-72^

The transition to resistance can be described with a net term of the form *εS* + *αu*(*t*)*S*. Mathematically, the drug-induced term *αu*(*t*)*S*, where *u*(*t*) is the effective applied drug dose at time *t*, describes the effect of treatment on promoting the resistant phenotype. For example, this term could represent the induced overexpression of the P-glycoprotein gene, a well-known mediator of multidrug resistance, by the application of chemotherapy.^1,73^

Spontaneous evolution of resistance is captured in the *ϵS* term, which permits resistance to develop even in the absence of treatment. Note that ε is generally considered small,^74^ although recent experimental evidence regarding error-prone DNA polymerases suggests that cancer cells may have increased mutation rates as a result of the overexpression of such polymerases.^75-77^ For example, in Krutyakov,^75^ mutation rates as a result of such polymerases are characterized by probabilities as high as 7.5 × 10^−1^ per base substitution, and it is known that many point mutations in cancer arise from these DNA polymerases.^77^ For this work, we adopt the notion that random point mutations that lead to drug resistance are rare, and that drug-induced resistance occurs on much quicker time scales^41^; therefore, we will assume that *α* > ε with *u* = *O*(1) in our analysis of Equations 1 and 2.

We model the effects of treatment by assuming the log-kill hypothesis,^78^ which states that a given dose of chemotherapy eliminates the same fraction of tumor cells regardless of tumor size. We allow for each cellular compartment to have a different drug-induced death rate (*d, d_R_*); however, to accurately describe resistance it is required that 0 ≤ *d_R_* < *d*. Our analysis presented herein will be under the simplest assumption that the drug is completely ineffective against resistant cells, so that *d_R_* = 0.

The last term in the equations, *γR*, represents the resensitization of cancer cells to the drug. In the case of nonreversible resistance, *γ* = 0; otherwise *γ* > 0. Our subsequent analysis will be done under the assumption of nonreversible resistance. For a discussion of the effect of reversibility on the presented model, see the Appendix.

Finally, we note that the effective drug concentration *u*(*t*) can be thought of as a control input. For simplicity, in this work we assume that it is directly proportional to the applied drug concentration; however, pharmacodynamic/pharmacokinetic considerations could be incorporated to more accurately describe the uptake/evolution of the drug in vivo or in vitro—for example, as in Bender, Schindler, and Friberg,^79^ Wu et al,^80^ and Fetterly et al.^81^

To understand the above system of drug resistance evolution, we reduce the number of parameters via nondimensionalization. Rescaling *S* and *R* by their (joint) carrying capacity *K*, and time *t* by the sensitive cell growth rate,

$$\begin{array}{l} \tilde{S}\left(\tau \right)=\frac{1}{K}S\left(\frac{1}{r}\tau \right),\\ \tilde{R}\left(\tau \right)=\frac{1}{K}R\left(\frac{1}{r}\tau \right), \end{array}$$ (3)

Equations 1 and 2 (with *γ* = *d_R_* = 0) can be written in the form,

$$\frac{dS}{dt}=\left(1-\left(S+R\right)\right)S-\left(\epsilon +\alpha u\left(t\right)\right)S-du\left(t\right)S,$$ (4)

$$\frac{dR}{dt}=p_{r}\left(1-\left(S+R\right)\right)R+\left(\epsilon +\alpha u\left(t\right)\right)S.$$ (5)

For convenience, we have relabeled S,R, and *t* to coincide with the nondimensionalization so that the parameters ε, *α*, and *d* must be scaled accordingly (by 1/*r*). As *r_R_* was assumed to satisfy 0 ≤ *r_R_* < *r*, the relative resistant population growth rate *p_r_* satisfies 0 ≤ *p_r_* < 1.

One can show (Appendix) that asymptotically, under any treatment regimen *u*(*t*) ≥ 0, the entire population will become resistant:

$$\left(\begin{array}{c} S\left(t\right)\\ R\left(t\right) \end{array}\right)\overset{t\to \infty }{\to }\left(\begin{array}{c} 0\\ 1 \end{array}\right).$$ (6)

However, tumor control is still possible where one can combine therapeutic efficacy and clonal competition to influence transient dynamics and possibly prolong patient life. Indeed, the modality of adaptive therapy has shown promise in using real-time patient data to inform therapeutic modulation aimed at increasing quality of life and survival times.^67^ This work will focus on such dynamics and controls.

## RESULTS

### Effect of Induction on Treatment Efficacy

We investigate the role of the induction capability of a drug (parameter α in Eqs 4 and 5) on treatment dynamics. Specifically, the value of α may have a substantial impact on the relative success of two standard therapy protocols—constant dosage and periodic pulsing.

### Treatment Protocol

**FIG 1.**
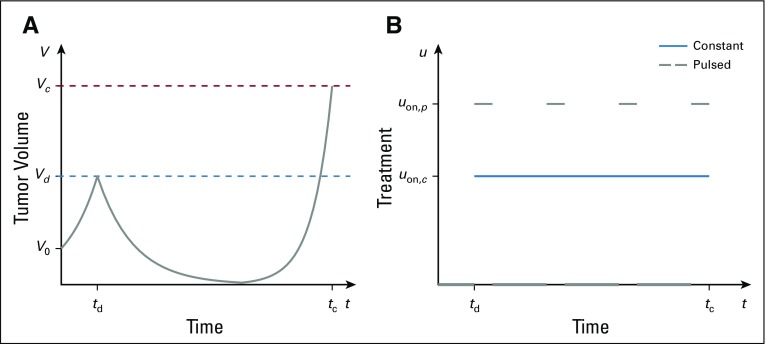
Schematic of tumor dynamics under two treatment regimes. (A) Tumor volume *V* in response to treatment initiated at time *t_d_*. Cancer population arises from a small sensitive population at time *t* = 0, upon which the tumor grows to detection at volume *V_d_*. Treatment is begun at *t_d_* and continues until the tumor reaches a critical size *V_c_* (at a corresponding time *t_c_*), where treatment is considered to have failed. (B) Illustrative constant and pulsed treatments, both initiated at *t* = *t_d_*.

To quantify the effects of induced resistance, a treatment protocol must be specified. We adopt a clinical perspective over the course of the disease, which is summarized in Figure 1. We assume that the disease is initiated by a small number of wild-type cells:

$$S\left(0\right)=S_{0},\;R\left(0\right)=0,$$ (7)

where 0 < *S_0_* < 1. Denote the tumor volume at time *t* by *V*(*t*):

$$V\left(t\right)=S\left(t\right)+R\left(t\right).$$ (8)

The tumor then progresses untreated until a specific volume *V_d_* is detected—or, for hematologic tumors, via appropriate blood markers—which using existing nuclear imaging techniques corresponds to a tumor with diameter on the order of 10 mm.^82^ Time to reach *V_d_* is denoted by *t_d_*, which in general depends on all parameters that appear in Equations 4 and 5. Note that, assuming *ϵ* > 0, a nonzero resistant population will exist at the onset of treatment. Therapy, represented through *u*(*t*), is then applied until the tumor reaches a critical size *V_c_*, which we equate with treatment failure. Because the (*S*,*R*) = (0,1) state is globally asymptotically stable in the first quadrant, *V_c_* < 1 is guaranteed to be obtained in finite time. Time until failure, *t_c_*, is then a measure of efficacy of the applied *u*(*t*).

Although a diverse set of inputs *u*(*t*) may be theoretically applied, presently we consider only strategies as illustrated in Figure 1B. The blue curve in Figure 1B corresponds to a constant effective dosage *u_c_*(*t*) initiated at *t_d_*—administered approximately using continuous infusion pumps and/or slow-release capsules—whereas the black curve represents a corresponding pulsed strategy *u_p_*(*t*), with fixed treatment windows (Δ*t*_on_) and holidays (Δ*t*_off_). In general, we may allow for different magnitudes, *u*_on,_*_c_* and *u*_on,_*_p_*, for constant and pulsed therapies respectively—for example, to relate the total dosage applied per treatment cycle (area under the drug concentration-time curve [AUC]^83^). However, for simplicity we assume the same magnitude in the subsequent section (although see the Appendix for a normalized comparison). While these represent idealized therapies, such *u*(*t*) may form an accurate approximation to in vitro and/or in vivo kinetics. Note that the response *V*(*t*) illustrated in Figure 1A will not be identical, or even qualitatively similar, for both presented strategies, as will be demonstrated numerically.

### Constant Versus Pulsed Therapy Comparison

To qualitatively demonstrate the role that induced resistance plays in the design of therapy schedules, we consider two drugs with the same cytotoxic potential—that is, the same drug-induced death rate *d*—each possessing a distinct level of resistance induction (parameter α). A fundamental question, then, is whether there exist qualitative distinctions between treatment responses for each chemotherapy. More specifically, how does survival time compare when scheduling is altered between constant therapy and pulsing? Does the optimal strategy—in this case, optimal across only two schedulings—change depending on the extent to which the drug induces resistance?

We fix two values of the induction parameter α:

$$\alpha_{s}=0,\;\;\;\;\;\;\;\;\;\;\;\;\;\alpha_{i}=10^{-2}.$$

Recall that we are studying the nondimensional model Equations 4 and 5, so no units are specified. Parameter *α* = 0 corresponds to no therapy-induced resistance (henceforth denoted as phenotype preserving), and therefore considering this case allows for a comparison between the classic notion of random evolution toward resistance (*α* = 0) and drug-induced resistance (*α* > 0). For the remainder of the section, parameters are fixed as in Table 1. Critically, all parameters excluding α are identical for each drug, which enables an unbiased comparison. Treatment magnitudes *u*_on,_*_c_* and *u*_on,_*_p_* are selected to be equal: *u*_on,_*_c_* = *u*_on,_*_p_* = *u*_on_.

**TABLE 1.**
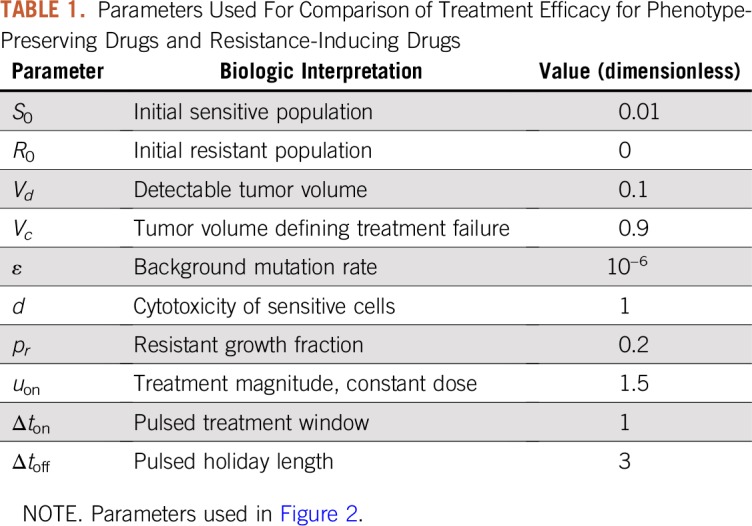
Parameters Used For Comparison of Treatment Efficacy for Phenotype-Preserving Drugs and Resistance-Inducing Drugs

Note that selecting parameter *V_d_* = 0.1 implies that the carrying capacity has a diameter of 100 mm, as *V_d_* corresponds to a detectable diameter of 10 mm. Assuming each cancer cell has volume 10^−6^ mm^3^, tumors in our model can grow to a carrying capacity of approximately 12.4 cm in diameter, which is in qualitative agreement with the parameters estimated in Chignola and Foroni^84^ (≈12.42 cm, assuming a tumor spheroid).

**FIG 2.**
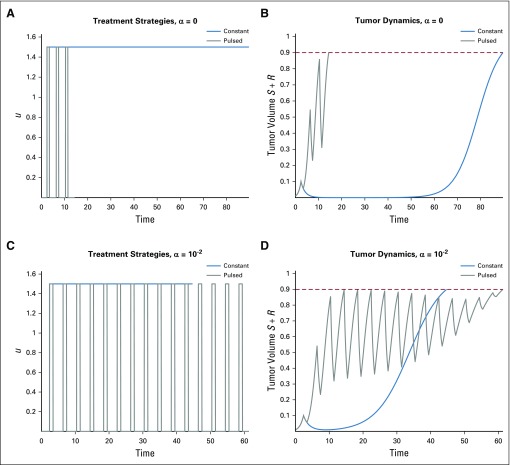
Comparison of treatment efficacy for phenotype-preserving drugs (*α* = 0) and resistance-inducing drugs (*α* = 10^−2^). The left column indicates treatment strategy, whereas the right column indicates corresponding tumor volume response. Note that the dashed red line in the right column indicates the tumor volume representing treatment failure, *V_c_*. (A) Constant and pulsed therapies after tumor detection for *α* = 0. (B) Responses corresponding to treatment regimens in panel A. (C) Constant and pulsed therapies after tumor detection for *α* = 10^−2^. (D) Responses corresponding to treatment regimens in panel C.

**FIG 3.**
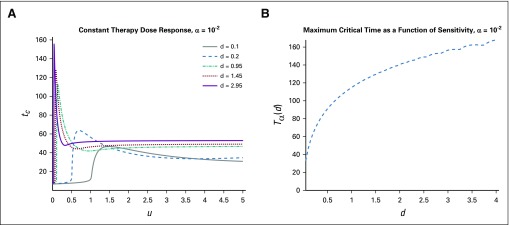
Variation in response time *t_c_* for a treatment that induces resistance. Constant therapy *u*(*t*) ≡ *u* is applied for *t_d_* ≤ *t* ≤ *t_c_*. Induction rate *α* = 10^−2^, with all other parameters as in Table 1. (A) Time until tumor reaches critical size *V_c_* for various drug sensitivities *d*. (B) Maximum response time *T_α_*(*d*) for a treatment that induces resistance. Note that time *T_α_*(*d*) increases with drug sensitivity; compare with Figure 4B for purely random resistance evolution.

**FIG 4.**
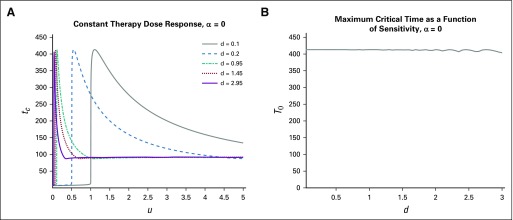
Change in critical time *t_c_* for differing drug sensitivities in the case of a phenotype-preserving treatment. (A) Time until tumor reaches critical size *V_c_* for various drug sensitivities *d*; comparable to Figure 3A, with *α* = 0. (B) Maximum critical time *T*_0_(*d*). Note that the curve is essentially constant.

**FIG A2.**
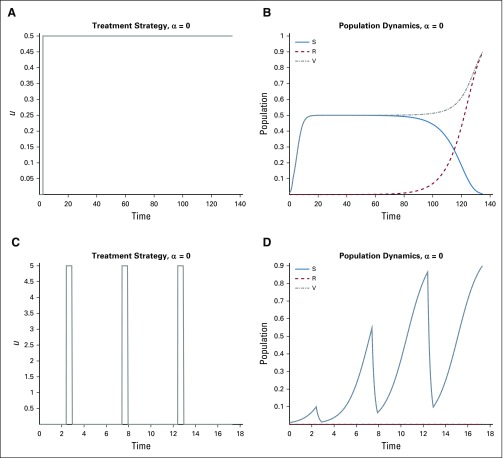
Treatment dynamics for phenotype-preserving drugs (*α* = 0). The left column indicates treatment strategy, whereas the right indicates corresponding population response. (A) Constant treatment after tumor detection. (B) Response to constant treatment. Note that at the time of treatment failure, the tumor is essentially entirely resistant. (C) Pulsed therapy after tumor detection. (D) Response to pulsed therapy. Note that the treatment fails much earlier versus the constant dose and that the tumor is primarily drug sensitive.

By examining Figures 2A and 2B, we clearly observe an improved response to constant therapy when using a phenotype-preserving drug, with treatment success time *t_c_* nearly seven times as long compared with pulsed therapy (*t_c_* ≈ 90 for constant, compared with *t_c_* ≈ 14 for pulsed). It can be observed that the tumor composition at treatment conclusion is different for each therapy—not shown for this simulation, but see a comparable result in Appendix Figures A2B and A2D—and it seems that pulsed therapy was not sufficiently strong to hamper the rapid growth of the sensitive population. Indeed, treatment failed quickly as a result of insufficient treatment intensity in this case, as the population remains almost entirely sensitive. Thus, for this patient under these specific treatments, assuming drug resistance only arises via random stochastic events, constant therapy would be preferred. One might argue that pulsed, equal-magnitude treatment is worse when *α* = 0 simply because less total drug—that is, AUC—is applied. However, we see that even in this case, intermediate doses may be optimal (Figs 3A and 4A). Thus, it is not the larger total drug, per se, that is responsible for the superiority of the constant protocol in this case, a point that is reinforced by the fact that the results remain qualitatively unchanged even if the total drug is controlled for (Appendix).

**FIG A3.**
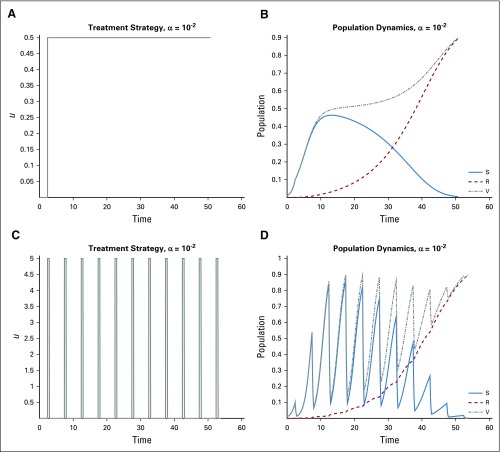
Treatment dynamics for resistance-inducing drugs (*α* = 10^−2^). The left column indicates treatment strategy, whereas the right indicates corresponding population response. (A) Constant treatment after tumor detection. (B) Response to constant treatment. Note that at the time of treatment failure, the tumor is essentially entirely resistant. Dynamics are similar to Figure 2B, although with a shorter survival time. (C) Pulsed therapy after tumor detection. (D) Response to pulsed therapy. Note that here, in contrast to the case of a phenotype-preserving drug as shown in Figure A2, pulsed therapy exhibits a longer survival time.

Compare this with Figures 2C and 2D, which consider the same patient and cytotoxicity, but for a highly inductive drug. Results are strikingly different and suggest that pulsed therapy is now not worse but in fact substantially improves patient response (*t_c_* ≈ 61 for pulsed, compared with *t_c_* ≈ 45 for constant). In this case, both tumors are now primarily resistant (Figs A3B and A3D), but the pulsed therapy allows for prolonged tumor control via sensitive/resistant competitive inhibition. Furthermore, treatment holidays reduce the overall flux into resistance as the application of the drug itself promotes this evolution. The total amount of drug (AUC) is also less for pulsed therapy (22.5 compared with ≈64), so that pulsed therapy is both more efficient in terms of treatment efficacy and less toxic to the patient as adverse effects are typically correlated with the total administered dose, which is proportional to the AUC. This is further consistent with recent experimental and clinical evidence that supports metronomic therapy as a superior alternative to classic chemotherapy regimens. The results presented in Figure 2 suggest that it may be advantageous to apply a smaller amount of drug more frequently; however, we also note that the results depend on patient-specific parameters, so that therapy would ideally be personalized to individual patients. Of note, we do not claim that these results hold for all parameter values—both patient and treatment specific—but instead emphasize that the rate of induction may play a large role in the design of therapies for specific patients.

For these specific parameter values, differences between constant and pulsed therapy for the inductive drug are not as extensive as in the phenotype-preserving case; however, recall that time has been nondimensionalized and, hence, the scale may indeed be clinically relevant. Such differences can be further amplified, and, as exact parameters are difficult or even (currently) impossible to measure, qualitative distinctions are paramount. Thus, at this stage, ranking of therapies, rather than their precise quantitative efficacy, should act as the more important clinical criterion.

From these results, we observe a qualitative difference in the treatment strategy to apply based entirely on the value of α, the degree to which the drug itself induces resistance. Thus, in administering chemotherapy, the resistance-promotion rate α of the treatment is a clinically significant parameter. In the next section, we use our model and its output to propose in vitro methods for experimentally measuring a drug’s α parameter.

### Identifying the Rate of Induced Drug Resistance

The effect of treatment on the evolution of phenotypic resistance may have a significant impact on the efficacy of conventional therapies. Thus, it is essential to understand the value of the induction parameter α before administering therapy. In this section, we briefly discuss both the theoretical possibility and practical feasibility of determining α from different input strategies *u*(*t*). For more details, see the Appendix.

### Theoretical Identifiability

We first study the structural identifiability of Equations 4 and 5, a prerequisite for analyzing practical methods for determining parameter values. Structural identifiability is the process of determining model parameters—for example, α—from a set of control experiments. Here, we assume that the only measurable quantity is the tumor volume *V* = *S* + *R*, along with its derivatives, in time. Using four different controls, we show that all model parameters, including the induction rate α, may be determined by precisely measuring the corresponding volume-response curves. For more details, see the Appendix.

### An In Vitro Experimental Protocol to Distinguish Spontaneous and Drug-Induced Resistance

As structural identifiability was established in the previous section, we focus on practical qualitative differences exhibited by Equations 4 and 5 as a function of the resistance-induction rate α. Utilizing only constant dosages, we investigate the dependence of *t_c_* on dose *u*, cytotoxicity *d*, and α. Defining the supremum over doses of the response time (Eq 8),

$$T_{\alpha }\left(d\right):=\underset{u}{\text{sup}}\left\{t_{c}\left(u,d,\alpha \right)\right\},$$ (9)

we plot the results for different α values in Figures 3 and 4.

**FIG 5.**
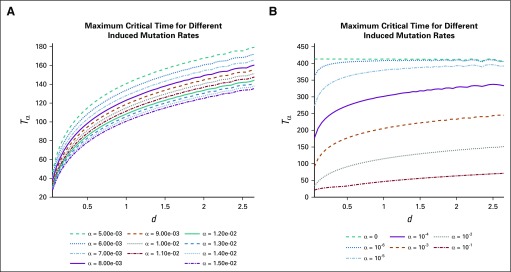
Variation in maximum response time for different induction rates α. For details on computation of *T_α_*(*d*), see Appendix Figure A4. All other parameters are given as in Table 1. (A) Plot of *T_α_*(*d*) for α near 10^−2^. (B) Analogous to panel A, where α is now varied over several orders of magnitude. Nonmutagenic case (*α* = 0) is included for reference.

Comparing Figures 3B and 4B, we observe a clear qualitative difference in maximum response times. In the case of a phenotype-preserving drug, the proposed in vitro experiment would produce a flat curve, whereas a resistance-inducing drug (*α* > 0) would yield an increasing function *T_α_* (*d*). Additional comparisons are presented in Figure 5, where the α dependence is more closely analyzed. For more details and analysis, see the Appendix.

## DISCUSSION

In the current work, we analyzed two distinct mechanisms that can result in drug resistance. Specifically, a mathematical model is proposed which describes both the spontaneous generation of resistance and drug-induced resistance. Using this model, we contrasted the effect of standard therapy protocols and demonstrate that contrary to the work in Liu et al,^34^ the rate of resistance induction may have a significant effect on treatment outcome. Thus, understanding the dynamics of resistance evolution with regard to the applied therapy is crucial.

To demonstrate that one can theoretically determine the induction rate, we performed an identifiability analysis on the parameter α and demonstrated that it can be obtained via a set of appropriate perturbation experiments on *u*(*t*). Furthermore, we presented an alternative method, using only constant therapies, for understanding the qualitative differences between purely spontaneous and induced cases. Such properties could possibly be used to design in vitro experiments on different pharmaceuticals, which allows one to determine the induction rate of drug resistance without an a priori understanding of the precise mechanism. We do note, however, that such experiments may still be difficult to perform in a laboratory environment, as engineering cells with various drug sensitivities *d* may be challenging. Indeed, this work can be considered as a thought experiment to identify qualitative properties that the induction rate α yields in our modeling framework.

Our simple model allows significant insight into the role of random versus induced resistance. Of course, more elaborate models can be studied by incorporating more biologic detail. For example, while our two-equation model classifies cells as either sensitive or resistant, not all resistance is treated equally. Some resistant cancer cells are permanently resistant, whereas others could transition back to a sensitive state.^41^ This distinction may prove to be vitally important in treatment design. A possible extension of our model is one in which we distinguish between sensitive cells *S*, nonreversible resistant cells *R_n_*, and reversible resistant cells *R_r_* (Eqs 9-12):

$$\frac{dS}{dt}=r\left(1-\frac{V}{K}\right)S-\left(\epsilon_{n}+\epsilon_{r}\right)S-\left(\alpha_{n}+\alpha_{r}\right)u\left(t\right)S-du\left(t\right)S+\gamma R_{r},$$ (10)

$$\frac{dR_{n}}{dt}=r_{n}\left(1-\frac{V}{K}\right)R_{n}+\epsilon_{n}S+\alpha_{n}u\left(t\right)S-d_{n}u\left(t\right)R_{n},$$ (11)

$$\frac{dR_{r}}{dt}=r_{r}\left(1-\frac{V}{K}\right)R_{r}+\epsilon_{r}S+\alpha_{r}u\left(t\right)S-\gamma R_{r}-d_{r}u\left(t\right)R_{r}.$$ (12)

Here, *V* denotes the entire tumor population—that is,

$$V:=S+R_{n}+R_{r}.$$ (13)

In this version of the model, nonreversible resistant cells *R_n_* can be thought of as resistant cells that form via genetic mutations. Under this assumption, *ε_n_* represents the rate at which spontaneous genetic mutations give rise to resistance, and *α_n_* is the drug-induced resistance rate. This situation can be classified as nonreversible as it is incredibly unlikely that genomic changes that occur in response to treatment would be reversed by an "undoing" mutation. Therefore, once cells confer a resistant phenotype via an underlying genetic change, we assume that they maintain that phenotype. This term could also be thought of as describing resistance that forms via stable epigenetic alterations or resistance that forms by some combination of genetic and stable epigenetic changes.

Conversely, reversible resistant cells *R_r_* denote resistant cells that form via phenotype switching, as described in Pisco et al.^41^ Random phenotype switching in the absence of treatment is captured in the ε*_r_S* term. This is consistent—and indeed necessary—to understand the experimental results in Pisco et al,^41^ where a stable distribution of MDR1 expressions is observed even in the absence of treatment. The *α_r_u*(*t*)*S* term represents the induction of a drug-resistant phenotype. Phenotype switching is often reversible, and therefore we allow a back transition from the *R_r_* compartment to the sensitive compartment at a nonnegligible rate γ^70^ (see Appendix). Formulated in this way, the model can be calibrated to experimental data and we can further consider the effects of the dosing strategy on treatment response. We plan to further study this model in future work.

Other extensions which include different clinical scenarios are also being investigated. In practice, chemotherapies are rarely applied in isolation. Multiple therapies are often administered simultaneously to improve efficacy. The inclusion of multiple drugs, including targeted therapies that act primarily on resistant subpopulations, yields natural control questions that are clinically relevant. Similarly, immune cells, together with immunotherapies, may also be incorporated to more accurately mimic the cancer microenvironment.

Overcoming drug resistance is crucial for the success of both chemotherapy and targeted therapy. Furthermore, the added complexity of induced drug resistance complicates therapy design, as the simultaneous effects of tumor reduction and resistance propagation confound one another. Mathematically, we have presented a clear framework for differentiating random and drug-induced resistance, which will allow for clinically actionable analysis on a biologically subtle, yet important, issue.

## Figures and Tables

**FIG A1.**
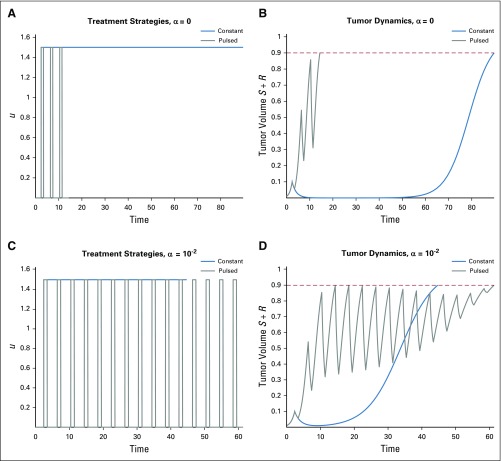
Comparison of treatment efficacy for phenotype-preserving drugs (*α* = 0) and resistance-inducing drugs (*α* = 10^−2^), where resistance is reversible. The left column indicates treatment strategy, whereas right indicates corresponding tumor volume response. Note that the dashed red line in the right column indicates the tumor volume representing treatment failure, *V_c_*. (A) Constant and pulsed therapies after tumor detection for *α* = 0. (B) Responses corresponding to treatment regimens in panel A. (C) Constant and pulsed therapies after tumor detection for *α* = 10^−2^. (D) Responses corresponding to treatment regimens in panel C.

**FIG A4.**
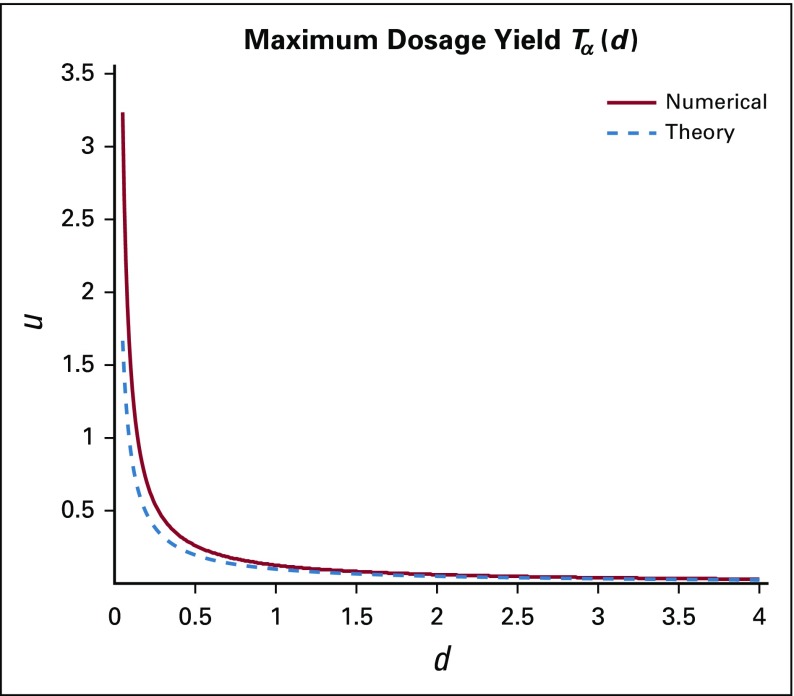
Dose yielding maximal response time *T_α_*(*d*) computed numerically, as well as the approximation given by Equation A24. All parameters appear as in Table 1, and *α* = 10^−2^. The numerical maximum is computed over a discretization of constant dosage procedures *u*(*t*) ≡ *u*, for u ∈ [0,5], with a mesh size Δ*u* = 0.005.

**FIG A5.**
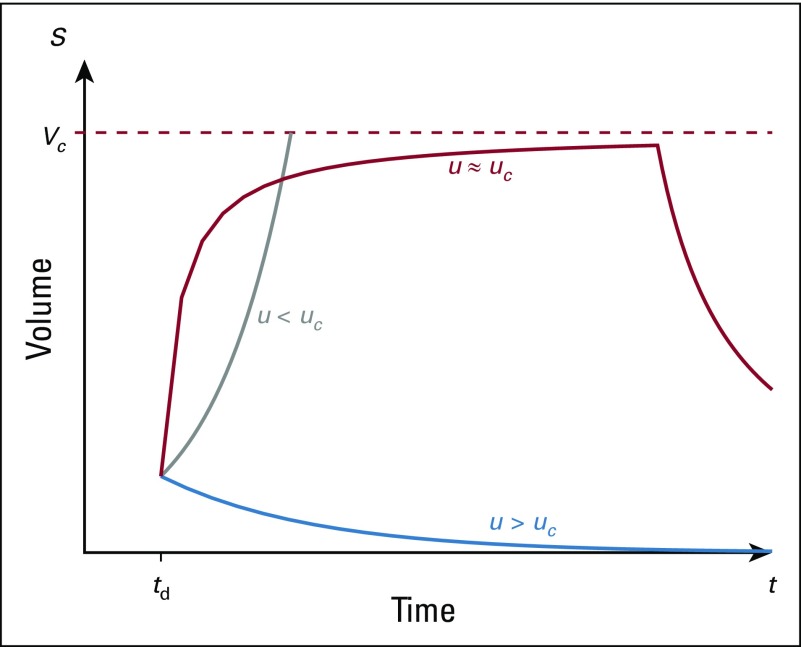
Schematic demonstrating dynamics of variation in *t_c_* on dosage *u*. Sensitive cell population plotted as a function of time for three representative doses. For *u* < *u_c_*, sensitive cells grow and reach *V_c_* in a short amount of time. As $u\to u_{c}^{-}$, the sensitive population approaches its approximate carrying capacity of *V_c_*, but subsequently decreases as a result of the dynamics of resistance. Here, *t_c_* is maximized as the sensitive population spends a large amount of time near *V_c_*. For *u* > *u_c_*, the sensitive population is eliminated quickly, and *V_c_* is obtained by a primarily resistant population.

## Data Availability

The following represents disclosure information provided by authors of this manuscript. All relationships are considered compensated. Relationships are self-held unless noted. I = Immediate Family Member, Inst = My Institution. Relationships may not relate to the subject matter of this manuscript. For more information about ASCO's conflict of interest policy, please refer to www.asco.org/rwc or ascopubs.org/jco/site/ifc. No potential conflicts of interest were reported

## Appendix

The supplement contains additional results and extensions related to the model Equations 1 and 2 presented in the main text. Sections include details on the mathematical characteristics of solutions of Equations 4 and 5, an extension describing the reversibility of drug resistance, treatment regimens with normalized dosages, details on structural identifiability, and an expanded discussion on the maximum critical time *T_α_*(*d*).

### Fundamental Solution Properties of Resistance Model

For convenience, Equations 4 and 5 are reproduced below:

$$\frac{dS}{dt}=\left(1-\left(S+R\right)\right)S-\left(\epsilon +\alpha u\left(t\right)\right)S-du\left(t\right)S,$$ (A1)

$$\frac{dR}{dt}=p_{r}\left(1-\left(S+R\right)\right)R+\left(\epsilon +\alpha u\left(t\right)\right)S.$$ (A2)

We begin with a standard existence/uniqueness result, as well as the dynamical invariance of the triangular region:

T:={(S,R)|S≥0,R≥0,S+R≤1}. (A3)

Note that *T* represents the region of non-negative tumor sizes less than 1. Biologically, this implies that all solutions remain physical (non-negative) and bounded above by the carry capacity (nondimensionalized to 1 here, generally *K* in Eqs 1 and 2).

#### Theorem 1.

For any bounded measurable control *u*: [0,∞) → [0,*u*_max_], with *u*_max_ < ∞, and (*S*_0_, *R*_0_) ∈ *T*, the initial value problem Equations A1 and A2,

$$S\left(0\right)=S_{0},\;\;R\left(0\right)=R_{0},$$

has a unique solution [*S*(*t*), *R*(*t*)] defined for all times *t* ∈ R. Furthermore, under the prescribed dynamics, region T is invariant.

*Proof.* Existence and uniqueness of local solutions follow from standard results in the theory of differential equations—for example (Sontag ED: Mathematical Control Theory: Deterministic Finite Dimensional Systems. Springer, 1998). As the vector field

$$F\left(S,R,t\right):=\left(\begin{array}{c} \left(1-\left(S+R\right)\right)S-\left(\epsilon +\alpha u\left(t\right)\right)S-du\left(t\right)S\\ p_{r}\left(1-\left(S+R\right)\right)R+\left(\epsilon +\alpha u\left(t\right)\right)S \end{array}\right).$$

is analytic for (*S*, *R*) ∈ R^2^, the existence of maximal solutions defined for all *t* ∈ R will follow from boundedness (Sontag ED: Mathematical Control Theory: Deterministic Finite Dimensional Systems. Springer, 1998), which we demonstrate below.

Uniqueness implies that solutions remain in the first quadrant for all *t* ≥ 0. Indeed, we first note that (0, 0) and (0 ,1) are steady states for any control *u*(*t*). As *S* = 0 implies that $\dot{S}=0$, we see that the *R*-axis in invariant, with $R\left(t\right)\overset{t\to \infty }{\to }1$. Similarly, *R* = 0 implies *Ṙ* ≥ 0, and hence all trajectories with (*S*_0_, *R*_0_) ∈ *T* remain non-negative for all *t*. As *V* = *S* + *R* satisfies the differential equation

$$\dot{V}=\alpha \left(t\right)\left(1-V\right)-\beta \left(t\right),$$

where *α*(*t*) := *S*(*t*) + *p_r_R*(*t*), *β*(*t*) := *du*(*t*)*S*(*t*) are both non-negative, $V_{0}<1\Rightarrow V\left(t\right)<1$. Thus, if the initial conditions (*S*_0_, *R*_0_) reside in *T*, we are guaranteed that (*S*(*t*), *R*(*t*)) ∈ *T* for all time *t*, as desired.

We now prove that, asymptotically, cells will evolve to become entirely resistant. For simplicity, we assume that the tumor is initially below carrying capacity, although a similar result holds for *V*_0_ > 1.

#### Theorem 2.

For any bounded measurable control *u: [0,∞) → [0,u_max_]* , with *u*_max_ < ∞, and initial conditions (*S*_0_, *R*_0_) ∈ *T*, solutions of Equations A1 and A2 will approach the steady state (*S*, *R*) = (0,1):

$$\left(S\left(t\right),R\left(t\right)\right)\overset{t\to \infty }{\to }\left(0,1\right).$$

*Proof.* From Theorem 1, we have that 0 ≤ *S*(*t*) + *R*(*t*) ≤ 1, so that Equation A2 implies

$$\frac{dR}{dt}\geq 0.$$

As 0 ≤ *R*(*t*) ≤ *S*(*t*) + *R*(*t*) ≤ 1, *R*(*t*) must converge, so that there exists 0 ≤ *R*_*_ ≤ 1 such that

$$R\left(t\right)\overset{t\to \infty }{\to }R_{*}.$$

Define

$$\rho :=\underset{t\to \infty }{\text{lim inf}}S\left(t\right).$$

Since *S*(*t*) ∈ [0,1], we know that 0 ≤ *ρ* ≤ 1. Assume that the inequality is strict on the left: *ρ* > 0. By definition, there then exists *t*_*_ > 0 such that

$$S\left(t\right)\geq \frac{\rho }{2},$$

for all *t* ≥ *t*_*_. As *S*(*t*) + *R*(*t*) ≤ 1 for all *t*, Equation A2 implies that for all *t* ≥ *t*_*_,

$$\dot{R}\left(t\right)\geq \epsilon S(t)\geq \frac{\epsilon \rho }{2},$$

which implies that

$$R\left(t\right)-R\left(t_{*}\right)\geq \frac{\epsilon \rho }{2}\int_{t_{*}}^{t}d\;s\overset{t\to \infty }{\to }\infty ,$$

a contradiction. Thus, *ρ* = 0.

Define

$$\sigma :=\underset{t\to \infty }{\text{lim inf}}\left(S\left(t\right)+R\left(t\right)\right).$$

As above, it is clear that *σ* ∈ [0,1]. Assume that *σ* < 1. Thus, there exists $t_{*},\bar{\epsilon }>0$ such that $S\left(t\right)+R\left(t\right)\leq 1-\bar{\epsilon }<1$ for all *t* ≥ *t*_*_. Equation A2 then implies that for all *t* ≥ *t*_*_,

$$\dot{R}\left(t\right)\geq p_{r}\left(1-\left(S\left(t\right)+R\left(t\right)\right)\right)R>p_{r}\bar{\epsilon }R,$$

As in the previous argument, this differential inequality contradicts the boundedness of *R*. Thus, *σ* = 1.

Since *R* converges to *R*_*_, lim inf distributes over the sum of *S* + *R*, and we have that

$$1=\underset{t\to \infty }{\lim\;\inf}\left(S\left(t\right)+R\left(t\right)\right)=\rho +R_{*}.$$

Thus *R*_*_ = 1.

As $R\left(t\right)\overset{t\to \infty }{\to }1$, there exists *t*_*_ > 0 such that

$$R\left(t\right)\geq 1-\frac{\epsilon }{2}$$

for all *t* ≥ *t*_*_. For such *t*, Equation A1 implies that *Ṡ*(*t*) < 0. Thus, *S* is eventually decreasing and hence must converge to its lim inf *ρ* = 0. Thus, we have that $\left(S\left(t\right),R\left(t\right)\right)\overset{t\to \infty }{\to }\left(0,1\right)$, as desired.

### Reversible Phenotype Switching

In the model analyzed in this work (Eqs 4 and 5), we assumed that resistance is nonreversible; however, experiments suggest^58^ that phenotypic alterations are generally unstable and hence a non-negligible back transition exists. In this Appendix, we demonstrate that an extension of our model to include this phenomenon does not change the qualitative results presented previously, at least for parameter values that are consistent with experimental data.

To model reversible drug resistance, we include a constant per-capita transition rate from the resistant compartment *R* back to the wild cell-type *S*. Denoting this rate by γ, we obtain the system

$$\frac{dS}{dt}=\left(1-\left(S+R\right)\right)S+\gamma R-\left(\epsilon +\alpha u\left(t\right)\right)S-du\left(t\right)S,$$ (A4)

$$\frac{dR}{dt}=p_{r}\left(1-\left(S+R\right)\right)R+\left(\epsilon +\alpha u\left(t\right)\right)S-\gamma R.$$ (A5)

We first consider an appropriate value of the rate γ. Note that in the absence of treatment (*u*(*t*) = 0), the system becomes

$$\frac{dS}{dt}=\left(1-\left(S+R\right)\right)S+\gamma R-\epsilon S$$ (A6)

$$\frac{dR}{dt}=p_{r}\left(1-\left(S+R\right)\right)R+\epsilon S-\gamma R.$$ (A7)

Note that the tumor volume *V*(*t*) = *S*(*t*) + *R*(*t*) satisfies the equation

$$\dot{V}=\left(S+p_{r}R\right)\left(1-V\right),$$ (A8)

which is nondecreasing (recall Theorem 1). This implies that the system approaches a steady state (*S*_*_, *R*_*_), which can be easily computed as

$$\left(S_{*},R_{*}\right)=\left(\frac{\gamma }{\gamma +\epsilon },\frac{\epsilon }{\gamma +\epsilon }\right).$$ (A9)

Note that Equation A9 lies on the line *V* = 1.

Pisco and colleagues^53^ measure a 1% to 2% subpopulation of clonally derived HL60 cells that consistently express high levels of MDR1, which we equate with the resistant population *R*. Using the 2% upper bound, this implies that

$$S_{*}=0.98,\;\;R_{*}=0.02.$$ (A10)

Solving Equation A9 with the above values then determines the ratio $\frac{\gamma }{\epsilon }$:

$$\frac{\gamma }{\epsilon }=49.$$ (A11)

We now repeat the constant versus pulsed experiments discussed in the main text, but for the reversible Equations A4 and A5. Parameter values are taken again as in Table 1, and γ is determined via Equation A11. Results are presented in Figure A1 and should be compared with that presented in Figure 2. Note that the same qualitative—and indeed quantitative—conclusions hold: constant therapy improves response time compared with pulsing when *α* = 0, whereas the reverse is true for *α* = 10^−2^. Thus, the inclusion of instability of the resistant cell subpopulation still suggests that knowledge of the resistance-induction rate α for a chemotherapy is critical when designing therapies. We note that precise agreement of Figures A1 and A2 is a result of the small values taken for ε and hence γ.

### Treatment Comparison for Equal Area Under the Drug Concentration-Time Curve

Here, we provide an analogous comparison of treatment outcomes between constant and pulsed therapy as in the main text; however, treatment magnitudes *u*_on,_*_c_* and *u*_on,_*_p_* are chosen such that

$$\int_{t_{d}}^{t_{d}+\Delta t_{\text{on}}+\Delta t_{\text{off}}}u_{c}\left(t\right)\text{d}t=\int_{t_{d}}^{t_{d}+\Delta t_{\text{on}}+\Delta t_{\text{off}}}u_{p}\left(t\right)\text{d}t,$$ (A12)

which is equivalent to the conservation of total administered dose between both strategies over a single pulsing cycle. In Equation A12, *u_c_*(*t*) and *u_p_*(*t*) denote constant and pulsed therapy schedules, respectively. The constant therapy magnitude *u*_on,_*_c_* is fixed (arbitrarily) at 0.5, which by Equation A12 implies that *u*_on,_*_p_* = 5. We also adjust Δ*t_on_* = 0.5, Δ*t_off_* = 4.5, and all other parameter values remain as in Table 1.

Results of the simulations are presented in Figures A2 and A3. Note that here Figure A2 displays the results for the phenotype-preserving drug (*α* = 0), whereas Figure A3 represents the resistance-inducing drug (*α* = 10^−2^). Each drug is simulated for the two distinct strategies; each strategy is represented in the left column of the respective figures. The right column illustrates the population response for both the individual populations—sensitive *S* and resistant *R* cells—as well as for the total tumor volume *V* = *S* + *R*. As previously discussed, treatment is continued until a critical tumor size *V_c_* is obtained, and the corresponding time *t_c_* is used as a measure of treatment efficacy, with a larger *t_c_* indicating a better response.

Our results are qualitatively in agreement with those presented in the main text (Fig 2), where no preservation of total administered drug (area under the drug concentration-time curve) was considered. Specifically, we observe a superior response with constant therapy for the phenotype-preserving drug (*α* = 0), whereas the situation is reversed for the resistance-inducing drug (*α* = 10^−2^).

### Identifiability

We provide details of both the structural and practical identifiability of control system Equations A1 and A2. Technical details are provided that do not appear in the main text.

#### Theoretical identifiability.

We first show that all parameters in Equations A1 and A2 are identifiable using a relatively small set of controls *u*(*t*) via classic methods from control theory. We provide a self-contained discussion. For a thorough review of theory and methods, see a recent article (Sontag ED: PLOS Comput Biol 13:e1005447, 2017) and the references therein.

Assuming that time and tumor volume are the only clinically observable outputs—that is, that one cannot readily determine sensitive and resistant proportions in a given population—we measure *V*(*t*) and its derivatives at time *t* = *t_d_* for different controls *u*(*t*). For simplicity, we assume that *t_d_* = 0, so that treatment is initiated with a purely wild-type (sensitive) population. Note that this is equivalent to assuming an entirely sensitive tumor at treatment initiation. Although the results remain valid if *t_d_* > 0 as the system of equations gain only a constant, this assumption will simplify the subsequent computations. For a discussion of the practical feasibility of such methods, see the next section.

Specifically, consider the Equations A1 and A2 with initial conditions (Eqn 7). Measuring *V*(*t*) = *S*(*t*) + *R*(*t*) at time *t* = 0 implies that we can identify *S*_0_:

$$V\left(0\right)=S\left(0\right)+R\left(0\right)=S_{0}=:Y_{0},$$

where we adopt the notation *Y_i_*,*i* ≥ 0 for measurable quantities. Similarly, define the following for the given input controls:

$$\begin{array}{l} Y_{1}\;:=V^{\prime }\left(0\right),\;u\left(t\right)\equiv 0,\\ Y_{2}\;:=V\prime \left(0\right),\;u\left(t\right)\equiv 1,\\ Y_{3}\;:=V\left(0\right),\;u\left(t\right)\equiv 0,\\ Y_{4}\;:=V\prime \prime \left(0\right),\;u(t)\equiv 1,\\ Y_{5}\;:=V\left(0\right),\;u\left(t\right)\equiv 2,\\ Y_{6}\;:=V^{\prime }\left(0\right),\;u\left(t\right)\equiv 0,\\ Y_{7}\;:=V^{\prime }\left(0\right),\;u\left(t\right)=t. \end{array}$$ (A13)

All quantities *Y_i_*,*i* = 0, 1,…, 7 are measurable, as each requires only knowledge of *V*(*t*) in a small positive neighborhood of *t* = 0. Note that the set of controls *u*(*t*) is relatively simple, with *Y*_7_ exclusively determined via a nonconstant input.

Each measurable *Y_i_* may also be written in terms of a subset of the parameters *d*, ε, *p_r_*, and α, as all derivatives can be calculated in terms of the right sides of Equations A1 and A2. For more details, see the below section. Equating the expressions yields a system of equations for the model parameter, which we are able to solve. Carrying out these computations yields the following solution:

$$d=-\frac{Y_{2}-Y_{1}}{Y_{0}},$$ (A14)

$$\epsilon =\frac{Y_{7}-Y_{6}+\frac{1}{2}Y_{5}-2Y_{4}-\frac{3}{2}Y_{3}+dY_{0}\left(1-Y_{0}\right)}{dY_{0}},$$ (A15)

$$p_{r}=\frac{Y_{3}-\left(1-\epsilon \right)Y_{0}+\left(3-\epsilon \right)Y_{0}^{2}-2Y_{0}^{3}}{\epsilon Y_{0}\left(1-Y_{0}\right)},$$ (A16)

$$\alpha =\frac{\frac{1}{2}Y_{5}-Y_{4}+\frac{1}{2}Y_{3}-d^{2}Y_{0}}{dY_{0}}.$$ (A17)

Note that in Equations A14 and A17 each quantity is determined by the *Y_i_* and the parameter values previously listed. We do not write the solution in explicit form for the sake of clarity as the resulting equations are unwieldy. Furthermore, the solution of this system relies on the assumption of strictly positive initial conditions (*S*_0_ = *Y*_0_ > 0), wild-type drug induction death rate (*d*), and background mutation rate (ε), all of which are made in this work.

Equation A17 is the desired result of our analysis. It demonstrates that the drug-induced phenotype switching rate α may be determined by a relatively small set of input controls *u*(*t*). As discussed in the previous section, the value of α may have a large impact on treatment efficacy and, therefore, determining its value is clinically significant. Our results now prove that it is possible to compute the induction rate and, hence, use this information in the design of treatment protocols. In the next section, we investigate other qualitative properties that could also be applied to understand the rate of drug-induced resistance.

### An In Vitro Experimental Protocol to Distinguish Spontaneous and Drug-Induced Resistance

We have demonstrated that all parameters in Equations 4 and 5 are identifiable so that it is theoretically possible to determine the phenotype-switching rate α from a class of input controls *u*(*t*); however, we see that the calculation involved measuring derivatives at the initial detection time *t* = *t_d_*. Furthermore, the applied controls (Eq A13) are nonconstant and thus require fractional doses to be administered. Clinically, such strategies and measurements may be difficult and/or impractical. In this section, we describe an in vitro method for estimating α using constant therapies only. Specifically, our primary goal is to distinguish drugs with *α* = 0 (phenotype preserving) and *α* > 0 (resistance inducing). Such experiments, described below, may be implemented for a specific drug, even if its precise mechanism of promoting resistance remains uncertain.

Before describing the in vitro experiment, we note that we are interested in qualitative properties for determining α. Indeed, in most modeling scenarios, we have little or no knowledge of precise parameter values and instead must rely on characteristics that distinguish the switching rate α independently of quantitative measurements. Furthermore, as a general framework for drug resistance, the only guaranteed clinically observable output variables are the critical tumor volume *V_c_* and the corresponding time *t_c_*. For a description of the treatment protocol, see above. We cannot expect temporal clonal subpopulation measurements. Assuming that *V_c_* is fixed for a given cancer, *t_c_* is thus the only observable that we consider.

By examining Equations 4 and 5, we see that the key parameters that dictate progression to the steady state (*S*, *R*) = (0,1) are *d* and α, as these determine the effectiveness and resistance induction of the treatment, respectively. Recall that ε is the fixed background mutation rate and *p_r_* the relative fitness of resistant cells. We thus perform a standard dose-response experiment for each value of drug sensitivity *d* and measure the time *t_c_* to reach critical size *V_c_* as a function of *d*. The response *t_c_* will then depend on the applied dose *u*—recall that we are only administering constant therapies—and the sensitivity of wild-type cells *d*, as well as the induction rate α:

$$t_{c}=t_{c}\left(u,d,\alpha \right).$$ (A18)

We further imagine that it is possible to adjust the wild-type drug sensitivity *d*. For example, in the case of multidrug resistance in which the overabundance of P-glycoprotein affects drug pharmacokinetics, altering the expression of MDR1 via ABCBC1 or even CDX2 (Koh I, Hinoi T, Sentani K, et al: Cancer Med 5:1546-1555, 2016) may yield a quantifiable relationship between wild-type cell and *d*, thus producing a range of drug-sensitive cell types. Figure 3A exhibits a set of dose-response curves for representative drug sensitivities *d* for the case of a resistance-inducing drug (*α* = 10^−2^).

For each of these cell types, we then define the supremum response time over administered doses:

$$T_{\alpha }\left(d\right):=\underset{u}{\text{sup}}\left\{t_{c}\left(u,d,\alpha \right)\right\}.$$ (A19)

Note that in a laboratory setting, only a finite number of doses will be administered so that the above supremum is actually a maximum, but for mathematical precision we retain supremum. Thus, we obtain a curve *T_α_* = *Τ_α_*(*d*) for each value of the induced resistance rate α. We then explore the properties of these curves for different α values.

Consider first the case of a phenotype-preserving drug, so that *α* = 0. As *u*(*t*) ≡ *u*, we see that the system Equations 4 and 5 depends only on the product of *u* and *d*. Hence, the dependence in Equation A18 becomes the form *t_c_*(*u*⋅*d*, 0), and thus the supremum in Equation A19 is instead across the joint parameter *D* := *u*⋅*d*:

$$T_{0}:=\underset{D}{\text{sup}}\left\{t_{c}\left(D,0\right)\right\}.$$ (A20)

Clearly, this is independent of *d* so that *T*_0_ is simply a horizontal line for *α* = 0. Qualitatively, the resulting curve will have no variation among the engineered sensitive phenotypes, save for experimental and measurement noise. Figure 4 shows both representative curves (Fig 4A compared with Fig 3A) and a plot of *T*_0_(*d*) (Fig 4B), which verifies its independence of *d*. We make two minor technical notes. First, it is important that we assume *d* > 0 here, as otherwise *D* = 0, independent of dose *u* and the supremum is over a one element set. See below for more details and the implications for α identifiability. Second, the slight variation for large values of *d* is a result of numerical error, as the maximum of *t_c_* occurs at decreasing doses (see the below section and Figure A4 for more details).

Comparing Figures 3A and 4A, we observe similar properties: small *t_c_* for small doses, a sharp increase about a critical *u_c_*, followed by smooth decrease and eventual horizontal asymptote (for mathematical justification, see the below section). However, note that for a resistance-inducing drug (Fig 3A), maximum critical time *T_α_*(*d*) increases as a function of *d*. This is in stark contrast to the constant behavior obtained for *α* = 0, argued above and demonstrated in Figure 4B. To further understand this phenomenon, we plot *T_α_*(*d*) for a fixed induction rate *α* = 10^−2^ in Figure 3B. The behavior of this curve is a result of the fact that the critical dosage *u_c_* at which *T_α_*(*d*) is obtained is a decreasing function of *d* (see Equation A24 and Fig A4 in the below section). But as *u_c_* also controls the amount of resistant cells generated—via the *αu*(*t*)*S* term—resistance growth is impeded by a decreasing *u_c_*. Thus, as a non-negligible amount of resistant cells are necessary to yield *T_α_*(*d*), more time is required for resistant cells to accumulate as *d* increases. Hence, *T_α_*(*d*) increases a function of *d*.

The behavior observed in Figures 3B and 4B is precisely the qualitative distinction that could assist in determining the induced resistance rate α. In the case of a phenotype-preserving drug, the proposed in vitro experiment would produce a flat curve, whereas a resistance-inducing drug (*α* > 0) would yield an increasing function *T_α_*(*d*). Furthermore, we could use this phenomenon, in principle, to measure the induction rate from the experimental *T_α_*(*d*) curve. For example, Figure 5A displays a range of *T_α_*(*d*) for α near 10^−2^.

Figure 5A shows a clear dependence of *T_α_*(*d*) on the value of α. Quantitatively characterizing such curves would allow us to reverse engineer the induction rate α; however, we note that, in general, the precise characteristics will depend on the other fixed parameter values, such as *p_r_*, *V_c_*, and *ε*. Indeed, only order of magnitude estimates may be feasible. Illustrative sample curves are provided in Figure 5B. Two such characteristics are apparent from this figure, both related to the slope of *T_α_*(*d*). First, as *d* → 0^+^, we observe an increase in the slope of *T_α_*(*d*) as α decreases (note that in Fig 5B, only *d* ≥ 0.05 are plotted). This follows from the continuity of solutions on parameters and the fact that *T*_0_(*d*) possesses a jump discontinuity at *d* = 0—that is, its distributional derivative is given by

$$\frac{\partial }{\partial d}|{}_{d=0}T_{0}\left(d\right)=k\delta \left(d\right),$$ (A21)

where δ is the Dirac function and *k* is a positive constant. As discussed previously (Equation A20 and the subsequent paragraph), *T*_0_(*d*) is flat, except at *d* = 0 where the vector field contains no *u* dependence. Therefore, the set over which the maximum is taken is irrelevant and *T*_0_(*d*) is thus proportional to the Heaviside function, which possesses the distributional derivative Equation A21. The constant *k* is determined by the size of the discontinuity of *T*_0_(*d*). Continuous dependence on parameters then implies that as α increases, the resulting derivative decreases away from positive infinity as the corresponding derivative for *T_α_*(*d*) with *α* > 0 is defined in the classic sense for *α* > 0:

$$\frac{\partial }{\partial \alpha }\frac{\partial }{\partial d}|{}_{d=0}T_{\alpha }\left(d\right)\leq 0.$$

The above argument implies that measuring the slope of *T_α_*(*d*) at *d* = 0 will give a characterization of the phenotypic alteration rate α of the treatment; however, such experiments may be impractical, as fine-tuning the sensitivity of a cell near complete resistance may be difficult. Alternatively, one could analyze the degree of flatness for a relatively large *d*—so to be sufficiently far from *d* = 0—and correlate this measure with α. For example, examining *d* = 2 in Figure 5B, we see that the relative slope of *T_α_*(*d*) with respect to *d* should correlate with decreasing α. An argument similar to the above makes this rigorous, for *d* sufficiently large. Practical issues still arise, but this second method provides a more global method for possibly computing α. Indeed, slopes at a given *d* can be approximated by a wider range of secant approximations as the result holds for a range of *d* compared with the previously discussed case when *d* is near zero. Furthermore, our focus is largely on the qualitative aspects of α determination, such as the differences in Figures 3A and 4A, and determining whether the treatment itself induces resistance to emerge.

### Introduction to Identifiability Analysis

In this section, we provide additional details on the theoretical identifiability of model parameters. As mentioned in the main text, all higher-order derivatives at initial time *t* = 0 may be calculated in terms of the initial conditions (*S*(0), *R*(0)) and the control function *u*(*t*). For example, for an arbitrary system

$$\dot{x}\left(t\right)=f\left(x\left(t\right),u\left(t\right)\right),$$

with external control *u*(*t*), the second derivative *ẍ* may be calculated using the chain rule:

$$\ddot{x}=J_{f}\left(x,u\right)f+\nabla_{u}f\left(x,u\right)\dot{u},$$

where *J_f_*(*x*) is the Jacobian matrix of *f*, evaluated at state *x* and control *u*. If *x*(0) = *x*_0_ is known, the above expression is a relation among parameters, together with *u* and $\dot{u}$ evaluated at time *t* = 0. An analogous statement holds for a measurable output *y* = *h*(*x*), but will also involve the Jacobian of *h*. Concretely, for the model of induced drug resistance Equations 4 and 5, first derivatives of the tumor volume may be calculated as

$$\begin{array}{c} V^{\prime }\left(0\right)=S^{\prime }\left(0\right)+R^{\prime }\left(0\right),\\ =\left(\left(1-\left(S+R\right)\right)S-\left(\epsilon +\alpha u\left(t\right)\right)S-du\left(t\right)S\right)+\left(p_{r}\left(1-\left(S+R\right)\right)R+\left(\epsilon +\alpha u\left(t\right)\right)S\right)|{}_{t=0},\\ =\left(1-S_{0}\right)S_{0}-du\left(0\right)S_{0}, \end{array}$$

for any control *u*(*t*) [recall that *R*(0) = 0]. Similarly, for the second derivative, we compute:

$$V^{\prime \prime }\left(0\right)=S_{0}\left(1-S_{0}-\left(\epsilon +d\right)u\left(0\right)\right)\left(1-2S_{0}-du(0)\right)+S_{0}\left(\alpha u\left(0\right)+\epsilon \right)\left(p_{r}-S_{0}\left(1+p_{r}\right)\right)-dS_{0}\dot{u}\left(0\right).$$

Using such expressions—or, more precisely, the Lie derivatives of the vector field [see Sontag (PLOS Comput Biol 13:e1005447, 2017)] —for the controls in Equation A13, one is able to obtain a set of equations between the set of *Y_i_*,*i* = 0, 1,…, 7 and the parameters *d*, *ε*, *p_r_*, and α. Solving these equations allows us to determine the parameters with respect to the measurable quantities. The algebraic solution is Equations A14-A17.

### Analysis of Critical Time *T_α_*(*d*)

We provide a qualitative understanding of the properties of *T_α_*(*d*), the maximum time, across all constant doses for the tumor to reach size *V_c_*. This Appendix is designed to explain the basic properties discussed in An In Vitro Experimental Protocol to Distinguish Spontaneous and Drug-Induced Resistance in the main text.

We first note that *T_α_*(*d*) is achieved at a medium dose *u_c_*. More precisely, we describe the qualitative properties of Figures 3A and 4A. Fix a drug sensitivity *d*. For small *u*, the sensitive subpopulation is not sufficiently inhibited and thus expands rapidly to cross the threshold *V_c_*, with an essentially homogenous population of sensitive cells. Indeed, as *u* → 0, the dynamics of Equations A1 and A2 approach those of

$$\frac{d\tilde{S}}{dt}=\left(1-\left(\tilde{S}+\tilde{R}\right)\right)\tilde{S},$$ (A22)

$$\frac{d\tilde{R}}{dt}=p_{r}\left(1-\left(\tilde{S}+\tilde{R}\right)\right)\tilde{R},$$ (A23)

for small finite times, as ε <<1. Trajectories of Equations A22 and A23 remain on the curve

$$R\left(S\right)=\frac{R_{0}}{S_{0}^{p_{r}}}S^{p_{r}}$$

as the solution approaches the line *S* + *R* = 1. The critical time *t_c_* is then determined by the intersection of this curve with *S* + *R* = *V_c_* and, thus, has sensitive population *S_c_* at *t_c_* given by the unique solution in the first quadrant of

$$S_{c}+\frac{R_{0}}{S_{0}^{p_{r}}}S_{c}^{p_{r}}=V_{c}.$$

As *R*_0_ << 1 (as ε <<1), *S_c_* ≈ *V_c_*, as claimed. Time *t_c_* is thus small for small *u*.

Increasing values of *u* imply that *t_c_* also increases as the overall growth rate of sensitive cells is decreased; however, there exists a critical dose *u_c_* such that sensitive cells alone are not able to multiply sufficiently to attain *V_c_*, so that the critical volume must have non-negligible contributions from the resistant fraction. This leads to the bifurcation apparent in Figures 3A and 4A. We can even approximate the critical dose maximizing *t_c_*, as *V_c_* must be an approximation for the carrying capacity of the sensitive cells:

$$S_{K}\approx V_{c}.$$

Examining the right side of Equation A1 and assuming that the dynamics of the resistant population are negligible, which is accurate in the early stages of treatment (Figs 2B and 3B), we see that the dose that yields the maximum temporal response should be

$$u_{c}\approx \frac{1-\epsilon -V_{c}}{\alpha +d}.$$ (A24)

That is, the dose at which *T_α_*(*d*) is obtained is given approximately by the expression in Equation A24. For a sample numerical comparison of the predicted formula Equation A24 and a numerical optimization over a range of drug sensitivities *d* (Figure A4). Note that in actuality, *S_K_* < *V_c_*, as the resistant dynamics cannot be ignored entirely. Thus, the precise value of *u_c_* will be smaller than that provided in the previous formula as we numerically observe. Lastly, *u_c_* decreases with increasing values of parameter *d* and, thus, requires an increasingly fine discretization to numerically locate the maximum value. Hence, some numerical error is observed in Figure 4B.

Lastly, as *u* is increased further, the dose becomes sufficiently large so that the inhibition of *S* via therapy implies that *S* cannot approach the critical volume *V_c_* and, hence, *V_c_* is again reached by an essentially homogeneous population, but now of resistant cells. As resistant cells divide at a slower rate (*p_r_* < 1), the corresponding time *t_c_* is smaller. For a schematic of the three regimes described above (Figure A5).
